# Novel Mutations Segregating with Complete Androgen Insensitivity Syndrome and Their Molecular Characteristics

**DOI:** 10.3390/ijms20215418

**Published:** 2019-10-30

**Authors:** Agnieszka Malcher, Piotr Jedrzejczak, Tomasz Stokowy, Soroosh Monem, Karolina Nowicka-Bauer, Agnieszka Zimna, Adam Czyzyk, Marzena Maciejewska-Jeske, Blazej Meczekalski, Katarzyna Bednarek-Rajewska, Aldona Wozniak, Natalia Rozwadowska, Maciej Kurpisz

**Affiliations:** 1 Institute of Human Genetics, Polish Academy of Sciences, 60-479 Poznan, Poland; agnieszka.malcher@igcz.poznan.pl (A.M.); soroosh.monem@gmail.com (S.M.); karolina.nowicka-bauer@igcz.poznan.pl (K.N.-B.); agnieszka.zimna@igcz.poznan.pl (A.Z.); natalia.rozwadowska@igcz.poznan.pl (N.R.); 2 Division of Infertility and Reproductive Endocrinology, Department of Gynecology, Obstetrics and Gynecological Oncology, Poznan University of Medical Sciences, 60-535 Poznan, Poland; piotrjedrzejczak@gmail.com; 3 Department of Clinical Science, University of Bergen, 5020 Bergen, Norway; tomasz.stokowy@k2.uib.no; 4 Department of Gynecological Endocrinology, Poznan University of Medical Sciences, 60-535 Poznan, Poland; czyzykadam@gmail.com (A.C.); marzena@jeske.pl (M.M.-J.); blazejmeczekalski@yahoo.com (B.M.); 5 Department of Clinical Pathology, Poznan University of Medical Sciences, 60-355 Poznan, Poland; juliaraj8@gmail.com (K.B.-R.); aldona.wozniak@wp.pl (A.W.)

**Keywords:** Morris syndrome, androgen insensitivity syndrome, infertility, spermatogenesis, biomarkers, rare disease, RNA-seq

## Abstract

We analyzed three cases of Complete Androgen Insensitivity Syndrome (CAIS) and report three hitherto undisclosed causes of the disease. RNA-Seq, Real-timePCR, Western immunoblotting, and immunohistochemistry were performed with the aim of characterizing the disease-causing variants. In case No.1, we have identified a novel androgen receptor (AR) mutation (c.840delT) within the first exon in the N-terminal transactivation domain. This thymine deletion resulted in a frameshift and thus introduced a premature stop codon at amino acid 282. In case No.2, we observed a nonsynonymous mutation in the ligand-binding domain (c.2491C>T). Case No.3 did not reveal AR mutation; however, we have found a heterozygous mutation in *CYP11A1* gene, which has a role in steroid hormone biosynthesis. Comparative RNA-Seq analysis of CAIS and control revealed 4293 significantly deregulated genes. In patients with CAIS, we observed a significant increase in the expression levels of *PLCXD3*, *TM4SF18*, *CFI*, *GPX8*, and *SFRP4,* and a significant decrease in the expression of *SPATA16, TSACC, TCP10L,* and *DPY19L2* genes (more than 10-fold, *p* < 0.05). Our findings will be helpful in molecular diagnostics of patients with CAIS, as well as the identified genes could be also potential biomarkers for the germ cells differentiation process.

## 1. Introduction

Androgen Insensitivity Syndrome (AIS, Morris syndrome, OMIM:300068) is one of the common causes of sex development disorders (DSDs). It affects -XY males but phenotypically is characterized by a female phenotype [1,2]. AIS presents with a wide spectrum of female features in individual patients with 46,XY karyotypes, ranging from Mild Androgen Insensitivity Syndrome (MAIS) to Partial Androgen Insensitivity Syndrome (PAIS) and to complete female phenotypes with external genitalia (Complete Androgen Insensitivity Syndrome; CAIS) [3]. The incidence of CAIS ranges from 1:20,000 to 1:64,000 of genetic males confirmed by molecular diagnosis [3,4]. The CAIS phenotype is identical to that of a normal female; however, features observed at puberty include the absence of menarche, absence of development of pubic and axillary hair development, a short blind-end vagina, absence of the uterus, and Müllerian duct degeneration. Moreover, testosterone levels rise earlier and are higher than in early pubescent men [5].

The AIS phenotype is caused by a reduced or absent functionality of the androgen receptor in both embryonic and secondary sexual development.

Normally, the male sexual development and reproductive functions are controlled by androgens, of which action is observed on the regulation of target genes [6] Androgens binding to androgen receptors (AR) leads to transactivation and binding of ARs to their response elements, which together with co-regulators promote the expression of target genes [7].

The main cause of AIS is AR mutations. The gene is located on the X chromosome, thus women with the 46,XX karyotype have no or minimal symptoms of androgen insensitivity. On the other hand, individuals with the 46,XY karyotype have more pronounced consequences of AR mutations. 

Currently, the Androgen Receptor Gene Mutations Database contains over 1000 recorded AR mutations including substitutions, deletions, insertions, duplications, and CAG expansions found throughout the 5′UTR, NTD, DBD, LBD, and 3′UTR [8]. Most of these mutations are located in the ligand binding domain and are inherited, although approximately 30% of cases feature de novo mutations. 

In 5% of individuals with AIS, causative mutation remains unknown [9]. Mutations in the other genes responsible for biosynthesis and metabolism of androgens and/or somatic mosaicism of an AR mutation are strongly suspected.

The aim of this study was to identify causative mutations in CAIS patients. We confirmed the genetic defects at the mRNA and protein levels, and identified genes potentially involved in AIS that regulate fertility and spermatogenesis.

## 2. Results

### 2.1. Identification of Mutations in AR and CYP11A1

**Figure 1:**
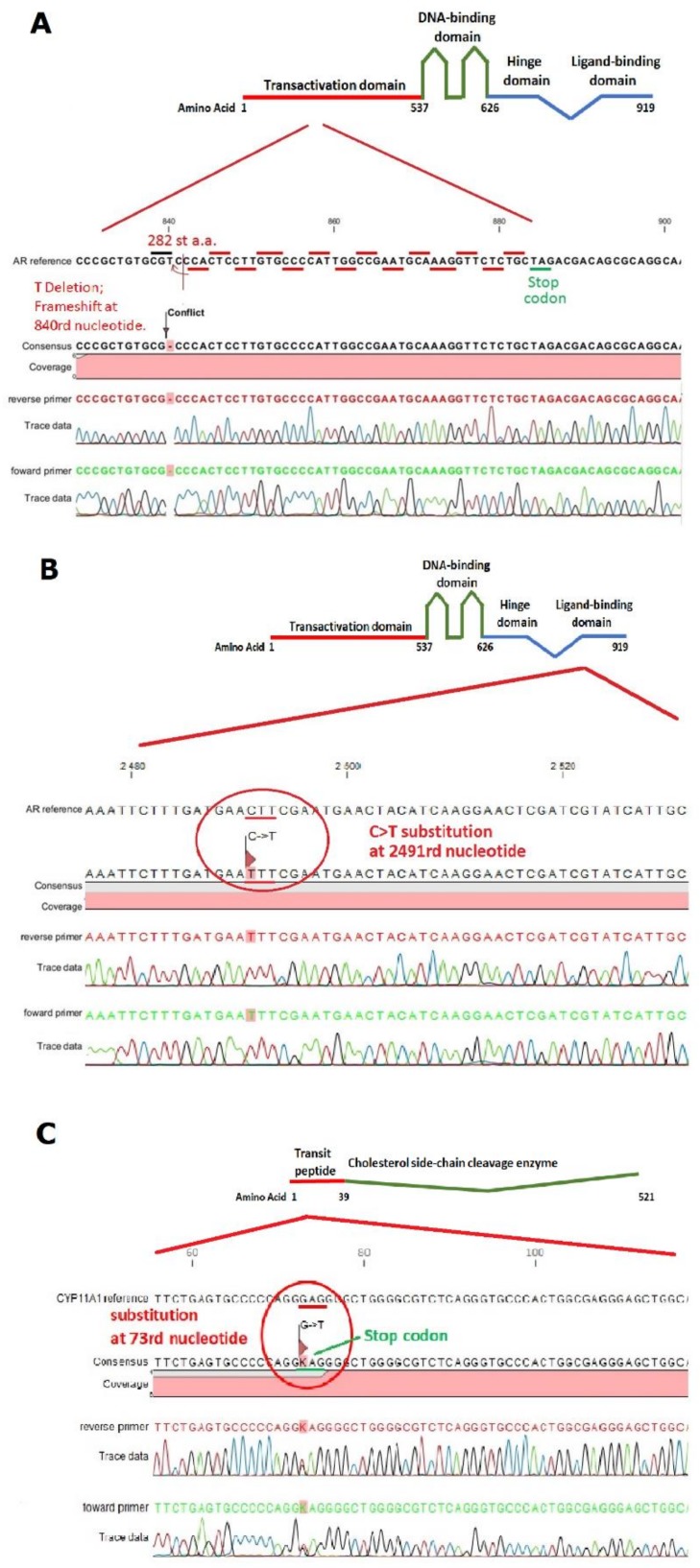
Identified mutations in Complete Androgen Insensitivity Syndrome (CAIS) patients: (**A**) Case No.1: Locus of the T deletion at the 840th nucleotide in the N-terminal transactivation domain (NTD) of AR, leading to a premature stop codon at the 282nd amino acid. (**B**) Case No.2: A C>T substitution which results in an amino acid change from Leucine (CTT) to Phenylalanine (TTT) at position 831 in AR. (**C**) Case No.3: The heterozygous G>T substitution (c.73G>T) results in a frameshift and introduction of a premature stop codon at amino acid 25 in CYP11A1. Red border indicate the localization in amino acid structure of the presented fragment. Red circle in Figure 1B,C indicate the nucleotide change in the presented fragment.

Case No.1: The AR sequence of this individual revealed a novel mutation within the first exon in the N-terminal transactivation domain (NTD). Thymine deletion in the NTD (c.840delT) resulted in a frameshift and introduction of a premature stop codon at amino acid 282 (Figure 1A).

Case No.2: The AR sequence of this individual revealed a mutation within the seventh exon in the ligand domain. The C>T substitution (c.2491C>T) resulted in an amino acid change from Leucine to Phenylalanine at position 831 (Figure 1B).

Case No.3: Sanger sequencing did not detect a mutation in the coding region of the AR gene. Therefore, we decided to use the RNA-seq results and identify the SNP (Single Nucleotide Polymorphism) variants, because in this case we only had the RNA material. Analysis of RNA-Seq data indicated a heterozygous mutation in the *CYP11A1* gene, which was subsequently confirmed by Sanger sequencing. The mutation was a G>T substitution (c.73G>T), which resulted in a frameshift and introduction of a premature stop codon at amino acid 25 referring to the reference sequence NM_000781 (Figure 1C).

### 2.2. Validation of AR and CYP11A1 Mutations at the mRNA and Protein Level in CAIS Cases

**Figure 2:**
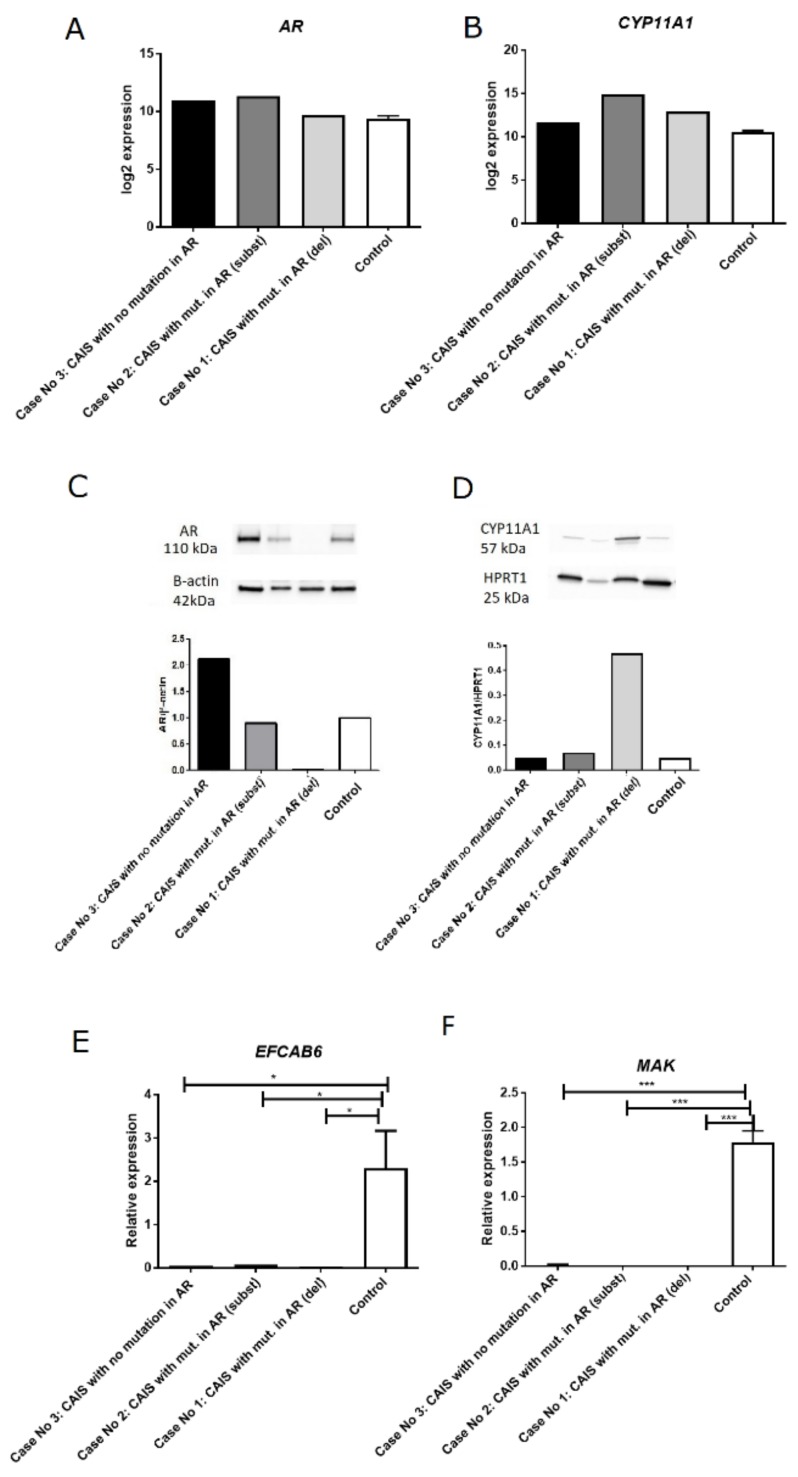
Validation of AR and CYP11A1 expression at the mRNA and protein levels. (**A**) Expression analysis of AR in patients with CAIS compared to men with normal spermatogenesis as analyzed by RNA-seq; (**B**) Expression analysis of CYP11A1A in patients with CAIS compared to men with normal spermatogenesis as analyzed by RNA-seq; (**C**) Western blot analysis of AR protein product and ACTB (reference protein); (**D**) Western blot analysis of CYP11A1 protein product and HPRT1 (reference protein); (**E**) Real-time PCR for EFCAB6; (**F**) Real-time PCR for MAK; the data are shown as relative expression, *p* < 0.05.

The expression of *AR* gene was higher (but not statistically significant) in Case No.2 and Case No.3 compared to the control group as measured by RNA-seq (Figure 2A). However, in the case with an identified mutation resulting in a deletion in AR (Case No.1), we did not detect the formation of androgen receptor protein by Western blot (Figure 2C). In Case No.2 (mutation with substitution in AR), we observed a slightly lower amount of AR protein (compared to the control), while in the Case No.3, we observed AR protein levels even in comparison to the control sample (Figure 2C). We also analyzed the expression level of *CYP11A1* and observed higher expression of this gene at mRNA level in Case No.1 and Case No.2 compared to men with normal spermatogenesis, as detected by RNA-seq (Figure 2B). However, at the protein level, the amount of CYP11A1 was similar to control samples, excluding the case with a nucleotide deletion in AR (case No.1) (Figure 2D). We also analyzed (by Real-Time PCR) two genes that were regulated by androgen receptor—*EFCAB6* and *MAK* (Figure 2E,F). We observed no expression of *EFCAB6* and *MAK* in all patients with CAIS compared to men with normal spermatogenesis. Even in case No.3, in which the AR protein is produced, these genes were not expressed (Figure 2E,F).

### 2.3. Immunohistochemistry (IHC)

**Figure 3:**
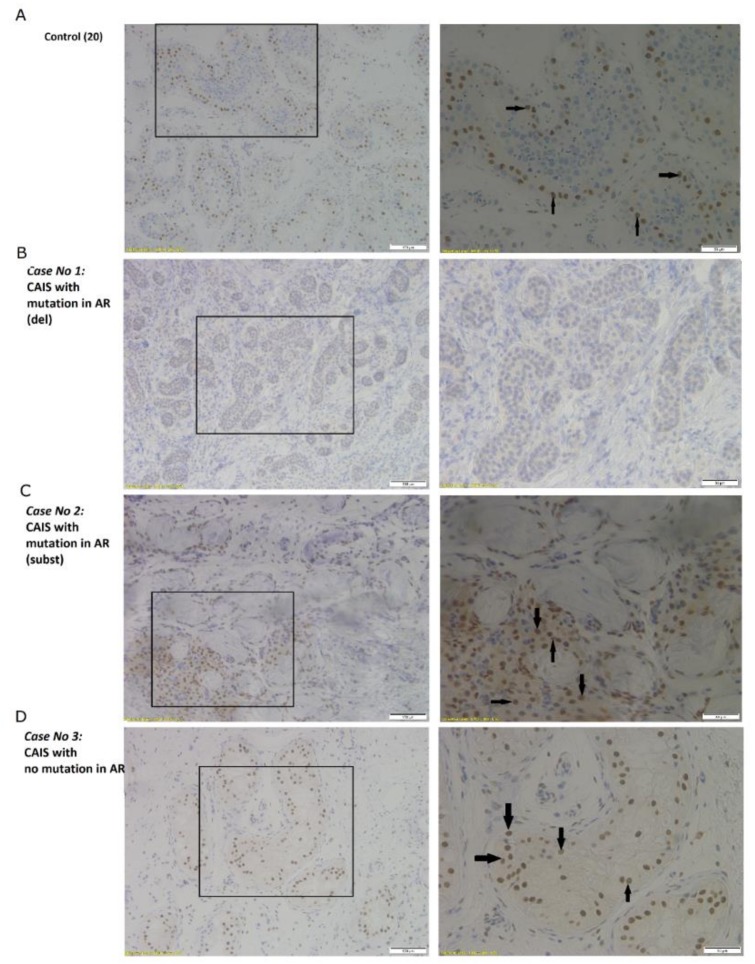
Immunohistochemistry of AR in: (**A**) men with normal spermatogenesis; (**B**) Case No.1: CAIS with no mutation in AR (del); (**C**) Case No.2: CAIS with mutation in AR (subst); (**D**) Case No.3: CAIS with mutation in AR. Scale bar: 100 µm (on the left) and 50 µm (on the right). Arrows indicate the AR positive staining. The black border indicate image presented on the right side.

**Figure 4:**
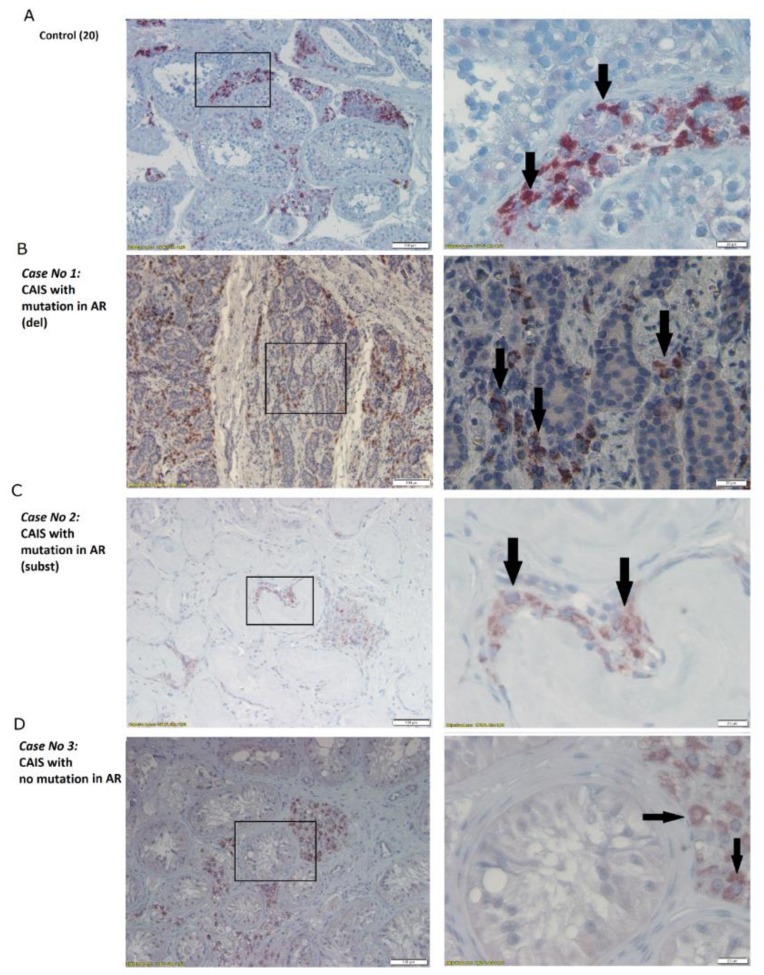
Immunohistochemistry of CYP11A1 in: (**A**) men with normal spermatogenesis; (**B**) Case No.1: CAIS with mutation in AR (del); (**C**) Case No.2: CAIS with mutation in AR (subst); (**D**) Case No.3: CAIS with no mutation in AR. Scale bar: 100 µm (on the left) and 20 µm (on the right). Arrows indicate the CYP11A1 positive staining.

We could clearly observe the lack of androgen receptor at the protein level in case No.1 in which we identified a deletion mutation in the *AR* (Figure 3B), whereas we noted localization of AR outside the seminiferous tubules in case No.2 with a substitution mutation in the *AR* sequence (Figure 3C). In case No.3 without a mutation in AR, we detected normally produced protein in the nucleus of Sertoli cells that was similar to in the control (Figure 3D). We also performed immunohistochemistry for CYP11A1, whose gene was identified as a heterozygous mutation in case No.3. However, in all the studied cases, we observed normal cytoplasmic staining of protein product in Leydig cells, even in case No.3 which contained the respective mutation (Figure 4).

### 2.4. Comparison of Gene Expression Profiles of Normal Versus Pathological Human Testis

**Figure 5:**
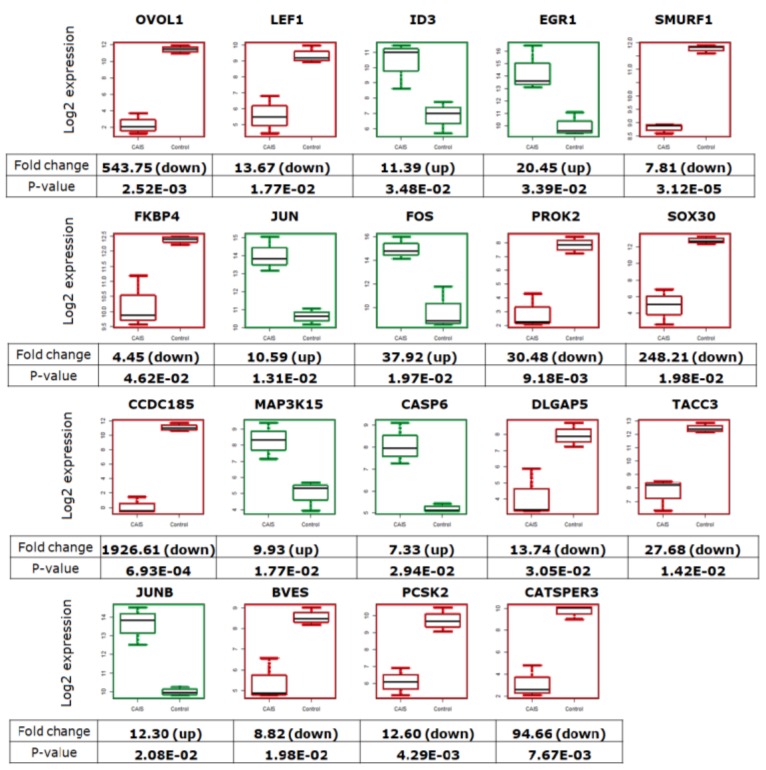
The box plots represent the sex development-related genes with differential expression in patients with CAIS compared to controls. Descriptions of the genes presented below demonstrate the fold change values in the box plots and the *p*-values. The green color indicates upregulation, while red indicates downregulation in patients with CAIS.

The comparative analysis of gene expression profiles by RNA-Seq between individuals with CAIS and controls (men with normal spermatogenesis) indicated 4293 differentially expressed genes with *p* < 0.05 (Table S1). These findings corroborate our earlier studies and showed that the gene expression profiles of patients with CAIS were significantly deregulated compared to controls. This deregulation is especially visible in genes closely related to sex development (Figure 5). In this study, we distinguished genes regulated/mediated by AR (*FKBP4*, *LEF1*, *EGR1*) and genes mostly associated with signal transduction of the MAPK or WNT pathway which are involved in sex differentiation (*SMURF1*, *MAP3K15, SOX30*, *PROK2).* We also observed significant upregulation of *ID3*, *EGR1*, *JUN*, *FOS*, *MAP3K15*, *CASP6*, and *JUNB* at a minimum 7-fold with *p* < 0.05 and downregulation of *OVOL1*, *LEF1*, *SMURF1*, *FKBP4*, *PROK2*, *SOX30*, *CCDC185*, *DLGAP5*, *TACC3*, *BVES, PCSK2*, and *CATSPER3* at minimum of 4-fold with *p* < 0.05 in patients with CAIS (Figure 5).

**Figure 6:**
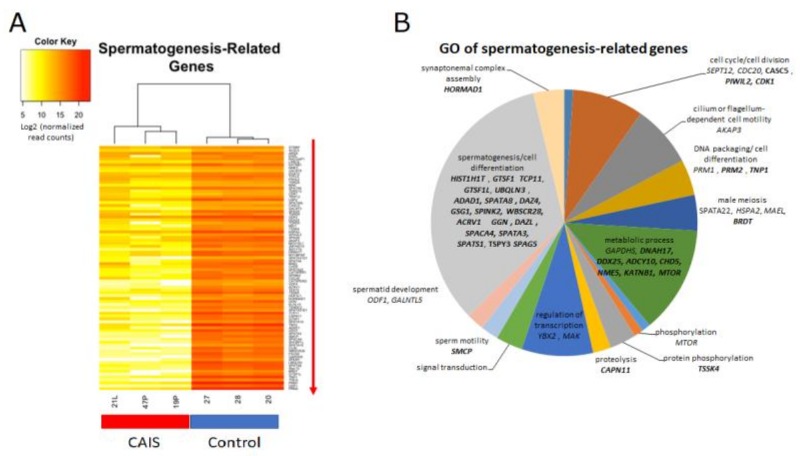
Transcriptomic analysis of spermatogenesis-related genes in patients with CAIS compared to the control group. (**A**) Heat map of spermatogenesis-related genes; (**B**) GO of selected spermatogenesis-related genes (Case No.1 = 19 P; Case No.2 = 47 P; Case No.3 = 21 L; men with normal spermatogenesis-20, 27, 28).

Genes involved in spermatogenesis, such as *PIWIL2*, *CAPN11*, *UBQLN3*, *GGN*, *SPATA3*, *SPACA4*, *SPATS1*, and protamines *PRM1* and *PRM2* were significantly downregulated in patients with CAIS compared to men with normal spermatogenesis (Figure 6). A detailed picture with expression levels of the majority of identified genes can be found in Figure S1.

**Figure 7:**
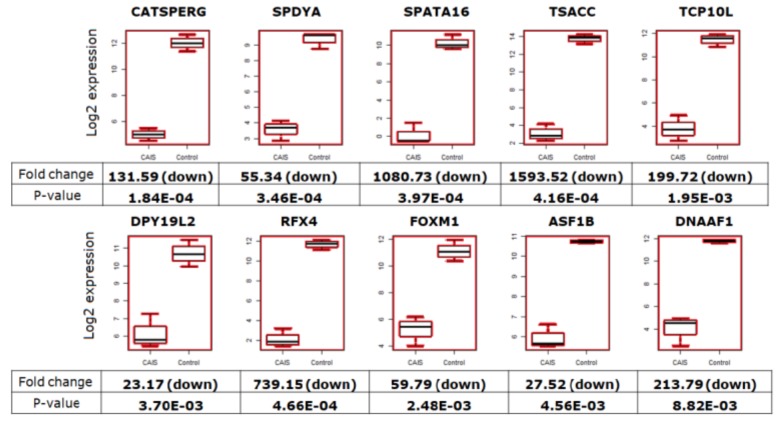
The box plots represent the fertility-related genes with downregulated expression in patients with CAIS compared to the control. Descriptions presented below the box plots demonstrate the fold change values and *p*-values. Red color indicate the downregulation of the genes in CAIS patients in comparison to the control

We also examined genes which have a direct impact on fertility and/or in which mutations were previously studied in animal models or human subjects by other research groups (Figure 7). These genes play important roles in cell proliferation (*RFX4, FOXM1, ASF1B*), differentiation during spermatogenesis (*CATSPERG*, *SPDYA*, *SPATA16*, *TSACC*, *TCP10L*, *DPY19L2*) and motility of sperm cells during fertilization (*DNAAF1*); mutations in these genes may lead to infertility. Expression levels of all of these genes were significantly downregulated at a minimum of 20-fold by *p* < 0.05 in patients with CAIS (Figure 7).

**Figure 8:**
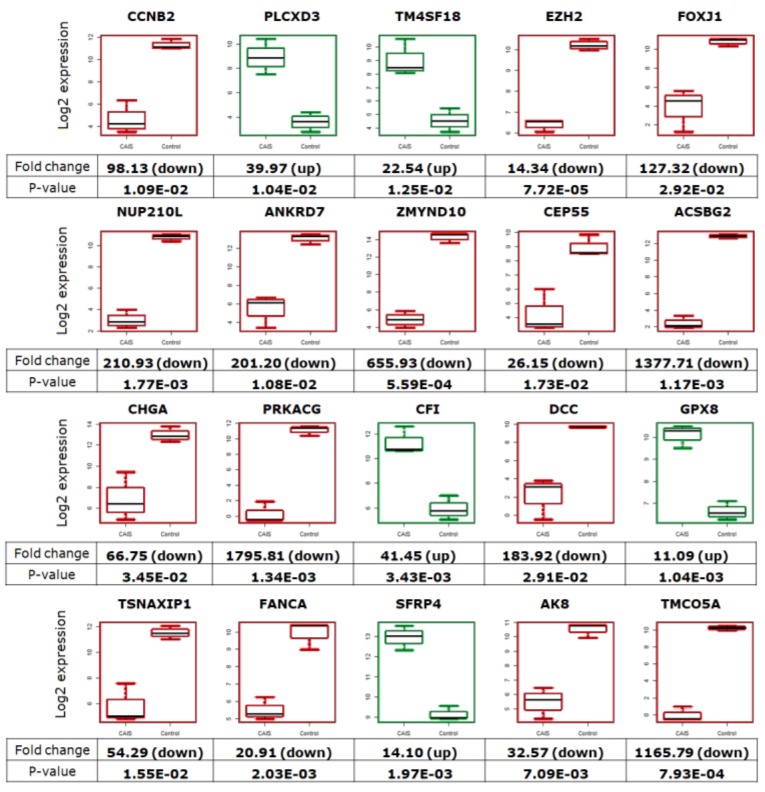
The box plots represent the genes with differentially expression in patients with CAIS compared to controls. Descriptions presented below the box plots demonstrate the fold change values and p-values. The green color indicates upregulation, and red indicates downregulation in patients with CAIS.

We also found genes which have never been studied before in connection to CAIS and spermatogenesis, and therefore their suspected function may be of great importance for maintaining reproductive ability. These genes presented different expression levels in all patients with CAIS in comparison to controls. *PLCXD3*, *TM4SF18*, *CFI*, *GPX8*, and *SFRP4* were upregulated at a minimum of 10-fold (*p* < 0.05), whereas *CCNB2*, *EZH2*, *FOXJ1*, *NUP210L*, *ANKRD7*, *ZMYND10*, *CEP55*, *ACSBG2*, *CHGA*, *PRKACG*, *DCC*, *TSNAXP1*, *FANCA*, *AK8*, and *TMCO5A* were downregulated (min. 14-fold, *p* < 0.05) in all patients with CAIS (Figure 8).

**Figure 9:**
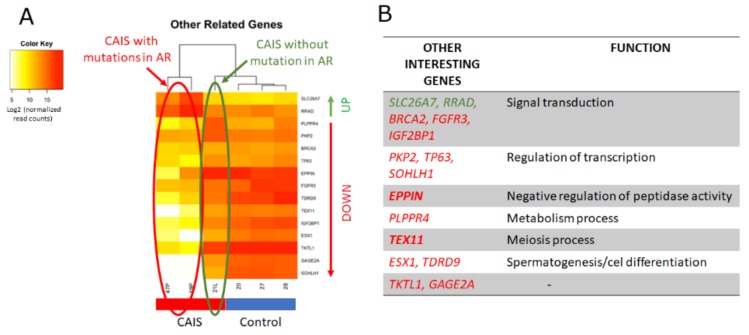
Genes with different expression levels in CAIS cases with mutations in AR (cases No.1 and 2) compared to the CAIS case without an AR mutation (case No.3) and the controls (genes marked in green indicate their upregulation and in red downregulation). (**A**) Heat map (**B**) Table presents the function of some identified genes (Case No.1 = 19 P; Case No.2 = 47 P; Case No.3 = 21 L; men with normal spermatogenesis-20, 27, 28).

Moreover, we also identified other genes with different expression levels only in individuals with *AR* mutations (cases No.1 and 2) compared to the CAIS individual without an AR mutation (case No.3) and controls. We selected genes mostly involved in signal transduction such as *SLC26A7* and *RRAD* which were upregulated and *BRCA2*, *FGFR3*, *IGF2BP1*, *PKP2*, *TP63*, *SOHLH1*, and a few directly involved in spermatogenesis such as *TEX11*, *ESX1*, and *TDRD9* which were downregulated in individuals with AR mutations (Figure 9). Unfortunately, we could not determine their statistical significance since we only analyzed a limited number of CAIS cases.

## 3. Discussion

This study includes molecular analysis of three cases of Complete Androgen Insensitivity Syndrome with a normal male karyotype. All three identified mutations are novel and so far have not yet been described. In the first case (case No.1), we identified a novel mutation: a thymine deletion in the region encoding the transactivation domain of androgen receptor, which led to a frameshift and introduced a premature stop codon (Figure 1A) causing significant shortening of the protein and probably its degradation, because we could clearly observe the lack of androgen receptor at the protein level by Western blot and IHC (Figure 2C and Figure 3B). In the second case (case No.2), we also identified a novel mutation in the ligand-binding domain; this was a C>T substitution which resulted in an amino acid change from Leucine (CTT) to Phenylalanine (TTT) (Figure 1B). This mutation shows the abnormal localization of AR, outside the seminiferous tubules (Figure 3C).

Similar examples have been documented in the existing literature, including both deletions and substitutions in the region of AR exon 1 or further exons encoding the ligand-binding domain. Most of them lead to complete or partial androgen insensitivity depending on the type of mutation [10,11,12,13,14,15]. However, the two presented cases (case No.1 and case No.2) revealed novel mutations that were not previously reported. Both cases lead to Complete Androgen Insensitivity Syndrome, and they contribute to our understanding of AIS and complement the androgen receptor gene mutation database (http://androgendb.mcgill.ca). 

In case No.3, we did not detect any mutations in the sequence fragment encoding the androgen receptor; however, we found a novel heterozygous mutation in the *CYP11A1* gene (Figure 1C), which was identified from the call of SNV variants by using RNA-Seq data. The mutation was a G>T substitution (c.73G>T) which resulted in a frameshift and introduction of a premature stop codon at amino acid 25 in one allele (Figure 1C). The majority of reported CAIS cases so far refer to mutations within the AR gene. It appears that only 5% of cases do not have mutation in this gene and the phenotypic variability in AIS can partly be explained by polymorphisms in genes responsible for the biosynthesis and metabolism of steroids [9]. In the literature, we found similar heterozygous mutations in *CYP11A1* [16,17,18]. *CYP11A1* encodes cytochrome P450scc, the mitochondrial cholesterol side chain cleavage enzyme, which is the only enzyme that catalyzes the conversion of cholesterol to pregnenolone and, thus, is required for the biosynthesis of all steroid hormones [16,19]. P450scc deficiency is a rare genetic disorder causing primary adrenal insufficiency with or without a 46,XY disorder of sexual development (DSD) [16]. 

In our case (case No.3) with a heterozygous mutation that introduces a premature stop codon at amino acid 25 in one allele, we observed that CYP11A1/P450scc is expressed at similar levels as in controls and individuals without CYP11A1 mutations, as detected by Western blot (Figure 2D). Additionally, the localization of the CYP11A1 protein product occurred in case No.3 was normal (Figure 4D). However, case No.3 had a lower testosterone level (2.09 ng/mL), which may be associated with the identified mutation in *CYP11A1* since the other cases (case No.1 and case No.2) had markedly higher testosterone levels (7.97 and 5.29 ng/mL, respectively) despite having a female phenotype. We also did not find any other mutations within the encoding region of the second *CYP11A1* allele. Therefore, the likely cause is the CYP11A1 insufficiency in 46,XY subjects with the E25* heterozygous mutation which is also consistent with the data received by Tajima et al. [18]. It is also possible that this mutation is supported by other mutation in regulatory regions of CYP11A1 or other mutations in genes responsible for the biosynthesis and metabolism of androgens. However, due to only RNA sample from this case, we were unable to conduct such studies.

In all three cases, the mutated variants were neither found in ExAC nor 1000G. These mutations are predicted as disease causing in the MutationTaster web application (http://www.mutationtaster.org/). In the MutationTaster it was distinguished: the amino acid sequence change and protein features were affected in all three cases. In case No.1, it also indicated the frameshift.

Moreover, we showed that two genes which directly interact with the androgen receptor- *EFCAB6* and *MAK* (Figure 2E,F) were not expressed, confirming dysfunction of the androgen receptor. In the case No.3 in which AR protein was produced similar to men with normal spermatogenesis (Figure 2C), we also observed lack of expression of *EFCAB6* and *MAK* (Figure 2E,F), what may be the result of identified heterozygous mutation in *CYP11A1* gene. This mutation may lead to the lower level of testosterone and may thus reduce the binding to the AR, and deactivate the AR signaling pathway. In consequence, *EFCAB6* and *MAK* will not be expressed. The *EFCAB6* gene, encodes the DJ-1-Binding Protein (DJBP), which is co-localized with AR in the nucleus through DNA-binding domains in DJ region. DJBP binds to the DBD of AR via DJ-1 and represses testosterone-dependent AR transactivation activity. Although this binding does not occur through DJBP’s common LXXLL motifs that are necessary to interact with nuclear receptors, DJBP is specifically expressed in the tests [20]. MAK is a kinase/phosphatase that enhances AR transcription through recruitment to the AR transcriptional complex in an androgen- and kinase-dependent manner. This co-activation of AR by MAK occurs in prostate tissue. MAK then phosphorylates other coactivators and chromatin proteins [21].

The most interesting part of these studies is that we established, for the first time, gene expression patterns clearly distinguishing patients with Complete Androgen Insensitivity Syndrome from men with normal spermatogenesis (Figure 5, Figure 6, Figure 7, Figure 8 and Figure 9). Our study shows that despite different genetic backgrounds of these CAIS individuals, the gene expression profiles are similar in each case when compared to the control group. Genes related to sex development were significant differently expressed in individuals with CAIS compared to men with normal spermatogenesis (Figure 5). We distinguished genes regulated/mediated by AR (*FKBP4*, *LEF1*, *EGR1*) and genes mostly associated with MAPK and WNT signal transduction such as *SMURF1*, *MAP3K15*, *SOX30*, and *PROK2* involved in sex differentiation [22,23,24].

We also showed that genes involved in spermatogenesis, such as *CAPN11*, *UBQLN3*, *GGN*, *SPATA3*, *SPACA4*, *SPATS1*, *PRM1*, *PRM2* [25], and other important genes involved in this process, were significantly downregulated in patients with CAIS in comparison to men with normal spermatogenesis (Figure 6), even in the Case No.3, where in single sections the germ cells and spermatogenesis were presented. Also genes in which mutations may lead to infertility (Figure 7), such as *DPY19L2* or *SPATA16,* were proved to be associated with male infertility in human globozoospermia studies [26,27,28].

Most importantly, we also found some intriguing genes which have never been studied in Androgen Insensitivity Syndrome and spermatogenesis, but their function and differential expression may be important to reproductive ability (Figure 8). For example, *TM4SF18* was over 22-fold upregulated in patients with CAIS in comparison to controls (Figure 8). *TM4SF18* has never been described; however, a member of this gene family was already investigated. *TM4SF* is highly expressed in testis and has regulatory effects on androgen receptor transactivation [29,30]; therefore, we may suggest that *TM4SF18* is also part of this complex. The *SFRP4* gene was also upregulated in individuals with CAIS (Figure 8). *SFRP4* is significantly enriched in the Wnt signaling pathway and is associated with apoptosis in rat ovulation, while the protein appears to antagonize a molecular pathway for cell survival [31].

Moreover, we also identified some other genes with different expression levels only in individuals with mutations in AR (case No.1 and case No.2) compared to the patient without an AR mutation (case No.3) and the control group (Figure 9). We highlighted genes involved in signal transduction, such as *SLC26A7* and *RRAD* which were upregulated and *BRCA2*, *FGFR3*, *IGF2BP1*, *PKP2*, *TP63*, *SOHLH1*, and others directly involved in spermatogenesis such as *TEX11*, *ESX1*, and *TDRD9* which were downregulated in patients with mutations in *AR* (Figure 9). These genes are worthy of attention because they may be directly regulated by *AR* and thus may help to understand the other genes action regulated by *AR*, which is still poorly understood in humans. On the other hand, it is worth to highlight the downregulated BRCA2 gene, which plays a role in DNA repair. This could be further associated with cancer development in later age. The risk of development of gonadal tumors may increase with age and were noted in 0.8–22% of the CAIS patients who have retained their gonads in adult life [32,33]. The most common presented testicular tumor in patients with CAIS was seminoma, which happened above the age of 30 [34]. However, due to the small number of studied individuals, these genes require further validation. An interesting approach would be also to further study the genes identified here not only in respect to novel potential biomarkers in CAIS patients, but also in patients with milder forms of AIS such as PAIS or MAIS.

## 4. Material and Methods

### 4.1. Subjects Studied

The biological material was collected from testicular biopsy specimens (3–5 mm^3^) of three patients preliminarily diagnosed with CAIS through clinical observations, gonadectomy and the standard procedure of testis histopathological evaluation. The human tissue collection procedure was approved by the Local Bioethical Committee at the Poznan Medical University (permission number 994/12; 08/11/2012) and the informed consent was obtained from each study participant. At the same time, we can confirm that all the experiments performed with human materials were in accordance with the relevant guidelines and regulations.

Case No.1: A 17-year-old girl. The cytogenetic studies demonstrated the 46,XY karyotype. Clinical examinations, based on the Quigley’s scale, showed features of Complete Androgen Insensitivity Syndrome. Hormonal analysis showed the following levels: FSH 2.81 mIU/mL, LH 13.88 mIU/mL, and Testosterone 7.97 ng/mL. Family anamnesis revealed no episodes of genetic diseases. The histopathological studies recognized tubular adenoma with immature seminiferous tubules without signs of spermatogenesis. Leydig cells were present in the testicular tissue.

Case No.2: A 44-year-old woman. Hormonal analysis showed the following levels: FSH 70.47 mIU/mL, LH 68.77 mIU/mL, and Testosterone 5.29 ng/mL. The patient had no relevant past medical or family history. The patient had no axillary hair and poorly developed secondary sex characteristics. The external genitalia appeared normal. The material prepared for histopathological analysis contained the structure of male gonads. The seminiferous tubules had Sertoli cells only. Many Leydig cells were visible in the testicular stroma. We observed a Sertoli-Leydig cell hamartoma in some regions. The entire microscopic picture corresponded to the clinical diagnosis of Complete Androgen Insensitivity Syndrome. More details regarding the clinical part of the case were published in a clinical presentation by Maciejewska-Jeske et al. [35].

Case No.3: A 14-year-old girl. The cytogenetic analysis revealed the 46,XY karyotype. The patient was clinically diagnosed as CAIS. Hormonal analysis showed the following levels: FSH N/A, LH 18.18 mIU/mL, and Testosterone 2.09 ng/mL. Testis structures were identified histopathologically. The Sertoli cells were present in the seminiferous tubules. In single sections, germ cells and spermatogenesis were observed. Abundant Leydig cells were visible. Nothing was known about the patient’s family history because the patient was a pupil of the Children’s Orphanage House.

### 4.2. Control Group

The entire transcriptome of the control group (*n* = 3) was obtained from commercial RNA sources obtained from men with normal testicular tissue with preserved spermatogenesis in age between 16–64 years (Clontech Laboratories; Agilent Life Technologies, Carlsbad, CA, USA; Ambion Life Technologies, Carlsbad, CA, USA). The control sample for Western Blot was obtained from commercial lysates prepared from normal testicular tissue (Novus Biologicals, Littleton, CO, USA).

### 4.3. RNA, DNA and Protein Extraction

The testicular samples were homogenized in 1 mL of TRI Reagent^®^ (Sigma-Aldrich, St. Louis, MO, USA), and RNA, DNA and protein were extracted according to the manufacturer’s protocol. The total RNA was post-purified using the RNeasy Plus Mini Kit to eliminate the DNA contamination (Qiagen, Hilden, Germany). At the end of the procedure, the protein was dissolved in 8 M urea, 50 mM Tris-HCl, pH 8.0 with 1% SDS (1:1) containing protease inhibitor cocktail (Roche, Basel, Switzerland).

### 4.4. DNA Sequencing

We have sequenced the coding region of AR for each patient studied. The reaction mixture for sequencing contained: 15–30 ng of DNA, 1 µL of AR primer (20 µM), 2 µL of BigDye (5x) buffer, and BigDye Terminator v3.1 (Applied Biosystems Life Technologies, Carlsbad, CA, USA) in a final volume of 20 µL. The primer pairs used for the sequencing reaction are listed in Table S2. The products were separated on an ABI Prism 310 (Applied Biosystems, Life Technologies, Carlsbad, CA, USA). Changes in the patient’s sequenced AR fragments were identified with respect to the reference DNA (made available in the NCBI database) using the CLC program Workbench 6.0. The AR sequence of each given individual were compared to the NCBI Reference Sequence NM_000044.3.

### 4.5. RNA Sequencing

Sequencing libraries were prepared from the total RNA (300 ng) according to the TruSeq RNA Sample Prep (Ver. A) (Illumina, San Diego, CA, USA) library protocol, and the high-throughput sequencing system, HiSeq (Illumina, San Diego, CA, USA) was used. The paired-end libraries had an average fragment size of 310 bp; the paired-end sequences were 100 bases in length. Expression data have been deposited in the Gene Expression Omnibus (GEO), accession number GSE125222.

### 4.6. Analysis of RNA-Seq Data

First, sequenced reads were aligned to the GRCh38.p7 reference genome using HISAT2 2.0.5 [36]. Variant calling of SNV variants for Case No.3 from RNA-Seq data was performed using samtools (REF), with the threshold of minimal 15x coverage. This approach allowed us to detect variants among genes with high and medium expression. Detected variants were annotated using Annovar (REF) and RareVariantVis [37]. Aligned reads within adequate GENCODE v25 gene annotation regions [38] were counted using featureCounts [39]. Read counts were further normalized using DESeq2 [40] in the R/Bioconductor environment [41].

### 4.7. Real-Time PCR

The cDNA was synthesized from 3 µg of total RNA using iScript™ Reverse Transcription Supermix (Bio-Rad Laboratories, Hercules, CA, USA) and 1.5 mL of 500 mM oligo d(T15) primers in a 20 µL reaction volume in the PTC-200 thermocycler (MJ Research, San Francisco, CA, USA). Real-time PCR was performed in a total volume of 25 mL using 2 mL of diluted cDNA (1:3), 12.5 mL of Supermix iQ SYBR Green (Bio-Rad Laboratories, Hercules, CA, USA), and 2.5 mL of each 4 mM specified primer. The information about primer sequences is summarized in Table S3. A standard curve was included as a positive control and to determine the reaction efficiency. The threshold cycle (*C*t) values of each studied transcript were analyzed with the CFX384 Touch™ Real-time PCR detection system (Bio-Rad Laboratories, Hercules, CA, USA) with the following conditions: initial denaturation at 95 °C for 60 s followed by 50 cycles at 95 °C for 20 s, 60 °C for 20 s, and 72 °C for 20 s. The threshold cycle (*C*t) values of each studied transcript were analyzed with the iCycler iQ5 Real-Time PCR Detection System (Bio-Rad Laboratories, Hercules, CA, USA). All samples and standard curves were run in duplicate. The relative expression level of each studied transcript was normalized to three housekeeping genes (*beta-actin*, *GAPDH*, and *HPRT1*) according to the GeNorm algorithm.

### 4.8. Western Blot

The total protein concentration was determined using the Lowry method. Fifty μg of protein was separated on 4–20% Mini-PROTEAN^®^ TGX Stain-Free™ Protein Gels (Bio-Rad Laboratories, Hercules, CA, USA) and electrotransferred to PVDF membranes (Bio-Rad Laboratories, Hercules, CA, USA) under standard conditions (30′) using a Trans-Blot^®^ Turbo (Bio-Rad Laboratories, Hercules, CA, USA). The membrane was soaked with blocking buffer containing non-fat milk (Bio-Rad Laboratories, Hercules, CA, USA). Immunodetection was performed using the antibodies listed in Table S4. Target proteins were detected by incubating the membrane with Clarity™ ECL Western Blotting Substrate (Bio-Rad Laboratories, Hercules, CA, USA) and analyzed with the ChemiDoc™ XRS system (Bio-Rad Laboratories, Hercules, CA, USA).

### 4.9. Immunohistochemistry

The formalin-fixed, paraffin-embedded sections presenting normal spermatogenesis were deparaffinized in xylene (2 × 10 min) and rehydrated 2 × 5 min in 100% ethanol, 96% ethanol, and 70% ethanol. Antigen retrieval was performed as follows: incubation in 2% NaBH_4_ for 30 min at room temperature, incubation for 30 min in 0.1% glycine at room temperature, and incubation in 0.01% sodium citrate solution at 95 °C for the next 30 min. Afterwards, the endogenous peroxidase was blocked with 3% H_2_O_2_ (in PBS) for 30 min. After rinsing with PBS, the histological section was blocked with 10% goat serum (in PBS) for 1 h and then incubated overnight with primary antibody (diluted in 1xPBS) at 4 °C. After rinsing 2 × 5 min with PBS, the section was incubated with secondary antibody (diluted in PBS) for 1 h at room temperature. Finally, the preparation was incubated with 4 drops of AEC Chromogen Kit (Sigma-Aldrich, St. Louis, MO, USA) for 10 min; after rinsing with H_2_O, the haematoxylin solution (Sigma-Aldrich, St. Louis, MO, USA) was applied for 1 min. The antibody dilutions are listed in Table S4.

### 4.10. Statistical Analysis

Differentially expressed genes were determined using the Student’s t-test with false discovery rate multiple testing correction (FDR). The median-based fold changes (MBFC) were calculated to evaluate the ratio of expression changes between the studied groups. A gene with MBFC > 2 or MBFC < 0.5 and FDR < 0.2 (adequate to Student’s t-test *p* value < 0.05) was considered significantly differentially expressed.

## 5. Conclusions

We identified novel mutations in the androgen receptor DNA sequence as well as in the *CYP11A1* gene in individuals with clinically diagnosed Complete Androgen Insensitivity Syndrome. Moreover, for the first time, we established a gene expression profile that clearly differentiates individuals with CAIS syndrome from men with normal spermatogenesis. Moreover, we showed that despite the different genetic backgrounds of CAIS, individuals with androgen insensitivity syndrome present a similar gene expression profile.

## Supplementary Materials

The following are available online at https://www.mdpi.com/1422-0067/20/21/5418/s1.
