# Supplementary figures and images for: Novel Mutations Segregating with Complete Androgen Insensitivity Syndrome and Their Molecular Characteristics
Source: Int J Mol Sci. 2019 Oct 30;20(21):5418. doi: 10.3390/ijms20215418 (PMC6861889; doi:10.3390/ijms20215418)

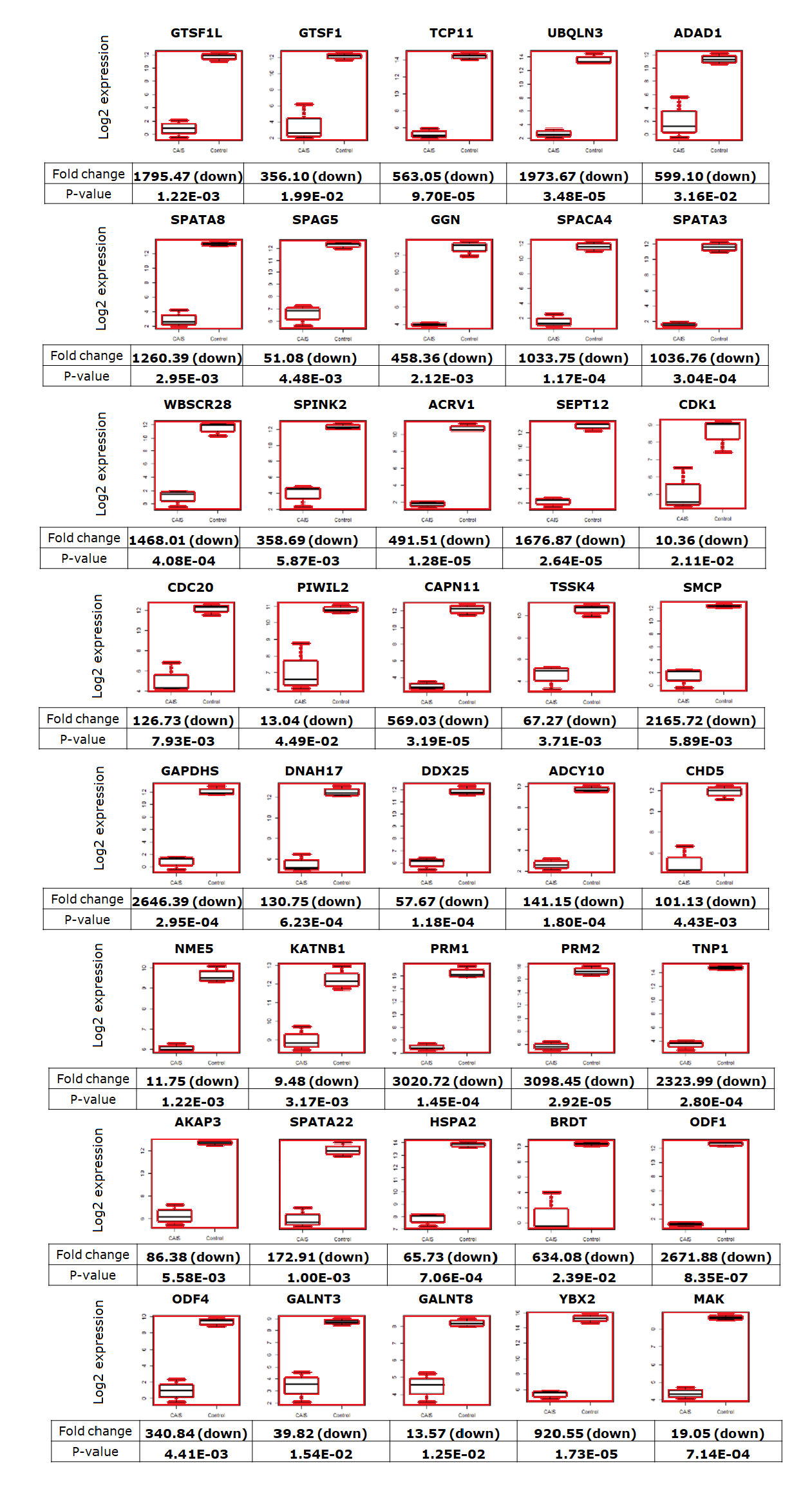

Supplement: Supplementary file 1 [file ijms-20-05418-s001.zip › ijms-626906-suppl/SupplementaryFigure 1.tif]
