# Supplementary material for: Novel Mutations Segregating with Complete Androgen Insensitivity Syndrome and Their Molecular Characteristics
Source: Int J Mol Sci. 2019 Oct 30;20(21):5418. doi: 10.3390/ijms20215418 (PMC6861889; doi:10.3390/ijms20215418)
Supplement: Supplementary file 1 [file ijms-20-05418-s001.zip › ijms-626906-suppl/Supplementary Table2.doc]

**Supplementary Table 2. The primer sequences used for DNA sequencing**

| Gene symbol | Primer | Primer sequence (5’🡪3’) | Product length | |
| --- | --- | --- | --- | --- |
| *AR_exon 1* | Forward | CATTTGCTCTCCACCTCCCAGCG | 628 | |
|  | Reverse | TCTCCGCGTGCAGCCTAACC |  |  |
|  | Forward | GGCTGCACGCGGAGAGAACC | 632 | |
|  | Reverse | TCACTTCGCGCACGCTCTGG |  |  |
|  | Forward | TCCAGAATCTGTTCCAGAGC | 287 | |
|  | Reverse | CTGTGAAGGTTGCTGTTCCTC |  |  |
|  | Forward | TCGTAGAGGCCCCACAGGCT | 542 | |
|  | Reverse | GACGCACAGCGGGTGGAACT |  |  |
|  | Forward | TGGGAGTTCCACCCGCTGTG | 809 | |
|  | Reverse | AAAAACTTACCGCATGTCCCCGT |  |  |
| *AR_exon 2* | Forward | GCCTGCAGGTTAATGCTGAAGACCT | 326 | |
|  | Reverse | TCCTGGGCCCTGAAAGGTTAGTGT |  |  |
| *AR_exon 3* | Forward | TGTTTGGTGCCATACTCTGTCCACT | 367 | |
|  | Reverse | GGCTGGTCCCCTTGGAAGCA |  |  |
| *AR_exon 4* | Forward | TCCCAATAGCCCGGAAGCTGA | 369 |  |
|  | Reverse | GTGGCTGGTCCATAGGAGCGT |  |  |
| *AR_exon 5* | Forward | ACCCAGACTGACCACTGCCTC | 201 |  |
|  | Reverse | GGCCCCAGGAGCACTTACTCA |  |  |
| *AR_exon 6* | Forward | CCTTCCCAGGTACCGCATGCAC | 200 |  |
|  | Reverse | GTGGTCCTCTCTGAATCTCTGTGCC |  |  |
| *AR_exon 7* | Forward | GGCATGCTTCCCCTCCCCATTC | 206 |  |
|  | Reverse | TGCTTACAGGCTGCACGGAGTC |  |  |
| *AR_exon 8* | Forward | AGGCTAGCAGAGGCCACCTCC | 806 |  |
|  | Reverse | TCGTCACTATTGGCCTCTTTGGGG |  |  |
| *CYP11A1 for cDNA* | Forward | ATGCTGGCCAAGGGTCTTC | 437 |  |
|  | Reverse | GCTGCCGACTTCTTCAACAG |  |  |
