# Supplementary material for: Novel Mutations Segregating with Complete Androgen Insensitivity Syndrome and Their Molecular Characteristics
Source: Int J Mol Sci. 2019 Oct 30;20(21):5418. doi: 10.3390/ijms20215418 (PMC6861889; doi:10.3390/ijms20215418)
Supplement: Supplementary file 1 [file ijms-20-05418-s001.zip › ijms-626906-suppl/Supplementary Table3.doc]

**Supplementary Table 3. Primer sequences for all target genes used in Real-Time PCR**

| **Gene ID** | **Primers** | **Primer sequences (5’->3’)** | **Product size (bp)** |
| --- | --- | --- | --- |
| *ACTB* | Forward | CTTCCTGGGCATGGAGTCC | 192bp |
|  | Reverse | ATCTTGATCTTCATTGTGCTG |  |
| *EFCAB6* | Forward | TGAGAGCAGTGGAGAACAGC | 156bp |
|  | Reverse | AAAATGCCGTGGACAAAGCC |  |
| *GAPDH* | Forward | GCTCTCTGCTCCTCCTGTTC | 112bp |
|  | Reverse | ACCAAATCCGTTGACTCCGA |  |
| *HPRT1* | Forward | CCTGGCGTCGTGATTAGTGA | 167bp |
|  | Reverse | TGATGGCCTCCCATCTCCTT |  |
| *MAK* | Forward | AAAGTGGTAGGAGGCGTTGG | 159bp |
|  | Reverse | GCTCTGGAAGCCGAAATGGA |  |
