# Supplementary material for: Novel Mutations Segregating with Complete Androgen Insensitivity Syndrome and Their Molecular Characteristics
Source: Int J Mol Sci. 2019 Oct 30;20(21):5418. doi: 10.3390/ijms20215418 (PMC6861889; doi:10.3390/ijms20215418)
Supplement: Supplementary file 1 [file ijms-20-05418-s001.zip › ijms-626906-suppl/Supplementary Table4.doc]

**Supplementary Table 4. Antibodies used for Western blot and immunohistochemistry**

| **Primary antibody** | | | **Secondary antibody** | | | |
| --- | --- | --- | --- | --- | --- | --- |
| **Name** | **Dilution** | **Application** | **Source** | **Name** | **Dilution** | **Source** |
| Anti-AR rabbit polyclonal antibody | 1:1000  1:50 | WB  IHC | Santa Cruz, Texas, USA | Goat anti-rabbit IgG-HRP | 1:40000  1:500 | Abcam, Cambridge, UK |
| Anti-CYP11A1 rabbit polyclonal antibody | 1:1000  1:500 | WB  IHC | Sigma-Aldrich, St. Louis, USA | Goat anti-rabbit IgG-HRP | 1:40000  1:500 | Abcam, Cambridge, UK |
| Anti-HPRT  rabbit polyclonal antibody | 1:10000 | WB | Abcam, Cambridge, UK | Goat anti-rabbit IgG-HRP | 1:40000 | Abcam, Cambridge, UK |
| Anti-ACTB mouse monoclonal antibody | 1:10000 | WB | Abcam, Cambridge, UK | Goat anti-mouse IgG-Peroxidase | 1:10000 | Sigma-Aldrich, St. Louis, USA |
